# Supplementary material for: Epidural Injection of Harpagoside for the Recovery of Rats with Lumbar Spinal Stenosis
Source: Cells. 2023 Sep 15;12(18):2281. doi: 10.3390/cells12182281 (PMC10526993; doi:10.3390/cells12182281)
Supplement: Supplementary file 1 [file cells-12-02281-s001.zip › cells-2574575-supplementary.pdf]

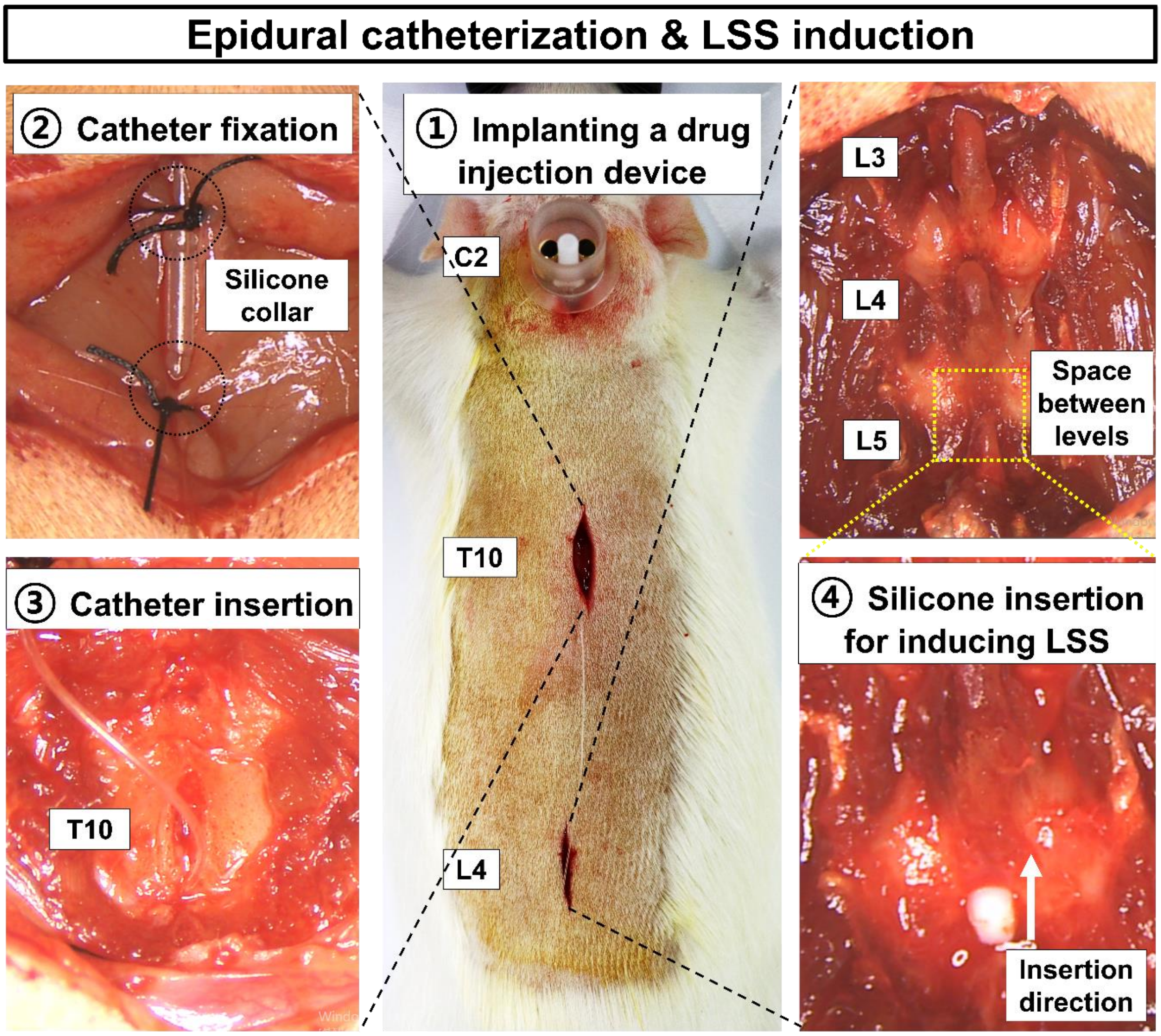

Figure S1. Images showing the surgical procedure of epidural catheterization and LSS induction. ① Implantation of drug injection device in C2 level. ② Fixation both sides of the silicone collar of the epidural catheter to the muscle. ③ Insertion of a catheter into the epidural space between the T10 and 11 levels. ④ Insertion of an 80 kpa silicone block into the spinal canal of L4 level for LSS induction.

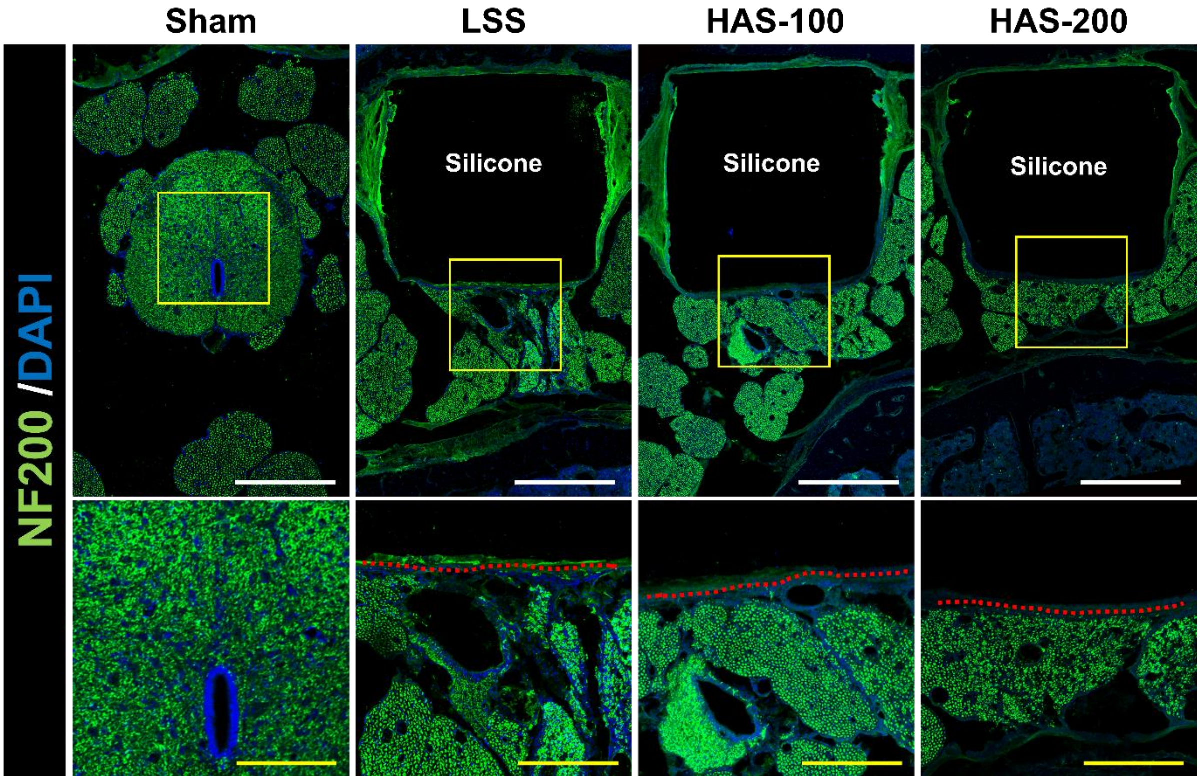

Figure S2. Representative IHC images of NF200 (green) in Sham, LSS, HAS-100, or HAS 200 groups. White scale bar = 500 μm, Yellow scale bar =200 μm.
